# Supplementary material for: SyNDI: synchronous network data integration framework
Source: BMC Bioinformatics. 2018 Nov 6;19:403. doi: 10.1186/s12859-018-2426-5 (PMC6219086; doi:10.1186/s12859-018-2426-5)
Supplement: Supplementary file 5 — Files of the example presented in Additional file 4. This additional file is a zip package that contains all files used in Additional file 4. Detailed descriptions of each of these files are presented in a README file included in this zip package. (GZ 30411 kb) [file 12859_2018_2426_MOESM5_ESM.gz › SyNDITools/Meme2Fimo/meme_4.9.0_4.tar.gz!/meme_4.9.0/doc/copyright.html]

```
Meta-MEME version 3.3 is copyright 1996-2007, William Stafford Noble,
Timothy L. Bailey and the Regents of the University of California.
All rights reserved.

Permission to use, copy, modify, and distribute any part of this
software for educational, research and non-profit purposes, without
fee, and without a written agreement is hereby granted, provided that
the above copyright notice, this paragraph and the following three
paragraphs appear in all copies.
                                                                
Those desiring to incorporate this software into commercial products
or use for commercial purposes should contact the Technology Transfer
Office, University of California, San Diego, 9500 Gilman Drive, La
Jolla, California, 92093-0910, Ph: (619) 534 5815.
                                                                
IN NO EVENT SHALL THE UNIVERSITY OF CALIFORNIA BE LIABLE TO ANY PARTY
FOR DIRECT, INDIRECT, SPECIAL, INCIDENTAL, OR CONSEQUENTIAL DAMAGES,
INCLUDING LOST PROFITS, ARISING OUT OF THE USE OF THIS SOFTWARE, EVEN
IF THE UNIVERSITY OF CALIFORNIA HAS BEEN ADVISED OF THE POSSIBILITY OF
SUCH DAMAGE.
                                                                
THE SOFTWARE PROVIDED HEREUNDER IS ON AN "AS IS" BASIS, AND THE
UNIVERSITY OF CALIFORNIA HAS NO OBLIGATIONS TO PROVIDE MAINTENANCE,
SUPPORT, UPDATES, ENHANCEMENTS, OR MODIFICATIONS.  THE UNIVERSITY OF
CALIFORNIA MAKES NO REPRESENTATIONS AND EXTENDS NO WARRANTIES OF ANY
KIND, EITHER EXPRESSED OR IMPLIED, INCLUDING, BUT NOT LIMITED TO, THE
IMPLIED WARRANTIES OF MERCHANTABILITY OR FITNESS FOR A PARTICULAR
PURPOSE, OR THAT THE USE OF THE MATERIAL WILL NOT INFRINGE ANY PATENT,
TRADEMARK OR OTHER RIGHTS.
```
